# Frequency of COVID-19 Infection as a Function of Vitamin D Levels

**DOI:** 10.3390/nu15071581

**Published:** 2023-03-24

**Authors:** Magdalena Basińska-Lewandowska, Krzysztof Lewandowski, Wojciech Horzelski, Andrzej Lewiński, Elżbieta Skowrońska-Jóźwiak

**Affiliations:** 1 “Your Family Doctor”, General Practice Surgery, 93-324 Lodz, Poland; mblbas@poczta.onet.pl; 2 Department of Endocrinology and Metabolic Diseases, Medical University of Lodz, 90-419 Lodz, Poland; krzysztof.lewandowski@umed.lodz.pl (K.L.); andrzej.lewinski@umed.lodz.pl (A.L.); 3 Department of Endocrinology and Metabolic Diseases, Polish Mother’s Memorial Hospital—Research Institute, 93-338 Lodz, Poland; 4 Department of Mathematics and Computer Science, University of Lodz, 90-238 Lodz, Poland; wojciech.horzelski@wmii.uni.lodz.pl

**Keywords:** vitamin D, COVID-19, disease prevention, healthy individuals

## Abstract

Background: It has been speculated that higher concentrations of 25-hydroxy-vitamin D (25OHD) provide some protection against COVID-19. We assessed whether there is any relationship between 25OHD concentrations and the subsequent development of COVID-19 infection. Materials and Methods: Concentrations of 25OHD were measured in March–April 2020 in 134 healthy subjects (57 males), age range 6–50, from a single urban general practice in central Poland. Data on COVID-19 infection during the subsequent 12 months (prior to the vaccination program) were obtained from the national database of COVID-19 cases. None of the subjects received any 25OHD supplements. Results: The average 25OHD concentrations were 18.1 ± 7.39 ng/mL (37.3% had 25OHD above 20 ng/mL). Thirty-one (23.1%) patients developed COVID-19 infection, but an increased risk was only observed in individuals with 25OHD concentrations below 12 ng/mL (COVID-19 infection in 11 out of 25 patients (44%) with 25OHD < 12 ng/mL versus 20 out of 109 (18.3%) for those with 25OHD above 12 ng/mL, *p* = 0.0063). Such a relationship was no longer observed for subjects with 25OHD concentrations above 20 ng/mL (*p* = 0.2787). Conclusions: Although only a minority of healthy subjects had 25OHD concentrations above 20 ng/mL in spring, an increased risk of subsequent COVID-19 infection was only observed in those with severe 25OHD deficiency (<12 ng/mL).

## 1. Introduction

The COVID-19 pandemic took the world by surprise; thus, prior to the instigation of the vaccination program, several measures were suggested to either reduce the risk of COVID-19 infection or decrease its severity. Vitamin D exerts several pleiotropic activities, including on the immune system, where, among other functions, it displays an anti-inflammatory effect, promotes dendritic cell and regulatory T-cell differentiation, and reduces T helper cell response and inflammatory cytokines secretion [1]. Hence, a role of vitamin D in the prevention and/or attenuation of the course of COVID-19 infection has been suggested [2,3]. Recently, in a large, retrospective, population-based study, improved COVID-19 outcomes in patients supplemented with cholecalciferol or calcifediol (*n* = 108,343), who achieved serum 25OHD levels ≥ 30 ng/mL, were shown. The group presented a lower risk of SARS-CoV2 infection, a lower risk of severe COVID-19, and lower COVID-19 mortality compared to 25OHD-deficient patients who were not receiving vitamin D supplements [4]. Moreover, some authors, e.g., Grant et al. [5], suggested that vitamin D supplementation aiming to increase 25OHD concentrations above 40–60 ng/mL (100–150 nmol/L) should provide significant protection against COVID-19, while some, e.g., Borsche et al. [6], even suggested that a mortality rate close to zero can be achieved with 25OHD concentrations above 50 ng/mL (125 nmol/L). As a result of such notions, patients were encouraged to take vitamin D supplements aiming for concentrations much higher than was necessary for bone health. In Poland, this was followed by a massive radio and TV advertisement campaign that encouraged healthy people to take vitamin D supplements in order to prevent COVID-19 and indeed all other infections.

On the other hand, some authors, such as Stroehlein et al. [7], stated that, “There is currently insufficient evidence to determine the benefits and harms of vitamin D supplementation as a treatment of COVID-19. The evidence for the effectiveness of vitamin D supplementation for the treatment of COVID-19 is very uncertain.” Hosseini et al. [8] claimed that 25OHD supplementation had no significant impact on the risk of COVID-19 infection, while recent VITAL study data [9] even questioned the use of vitamin D supplements for fracture prevention, demonstrating that vitamin D3 supplementation did not result in a significantly lower risk of fractures than placebo among generally healthy midlife and older adults who were not selected for vitamin D deficiency, low bone mass, or osteoporosis. Some authors [10] suggested that more research was needed to assess the impact of 25OHD supplementation for the treatment of patients with COVID-19, while Rubin R [11] nicely summarized the uncertainty regarding the relationship between vitamin D deficiency and COVID-19 risk, also discussing potential conflict of interests, e.g., related to the sponsoring of some studies by companies directly involved in vitamin D testing and/or supplementation.

On the strength of that, we decided to assess whether there was any relationship between 25OHD status at the beginning of the COVID-19 pandemic and the subsequent contraction of COVID-19 in unvaccinated healthy individuals who did not take any vitamin D supplements. In such a way, we also attempted to address the question commonly asked in general practice of whether patients should take vitamin supplements in order to obtain high 25OHD concentrations, which, theoretically, should protect them against COVID-19 infection.

## 2. Materials and Methods

This was a retrospective observational study that included 134 subjects (57 males) age 29.88 ± 14.37 (mean ± SD), range 6–50 years, body mass index (BMI) 25.32 ± 6.33 kg/m^2^. We chose this age range as both young children as well as individuals above 50 (particularly perimenopausal women) tend to take vitamin D supplements, while we aimed to assess the frequency of COVID-19 infection in a supplement-free population. There were no sex-related differences in BMI (25.09 ± 6.11 kg/m^2^ versus 25.57 ± 6.53 kg/m^2^) or age (26.82 ± 13.34 years versus 30.39 ± 14.53 years, for males and females, respectively). Concentrations of 25OHD vitamin D (25OHD), parathormone (PTH) and total calcium were assessed in March–April 2020. All subjects had normal kidney function, no history of liver disease, were not vegetarians, and were not taking any vitamin D supplements. Pregnant or breast-feeding women were not included in the study. All subjects were also free of any major disabilities that could impede their stay outdoors. After obtaining their informed consent, all investigated individuals were recruited from a single general practice in the city of Lodz (Poland). Cases of COVID-19 infection were obtained from a state database of COVID-19 cases, which could be accessed through the webpage www.gabinet.gov.pl (accessed on 1 June 2020), where confidential patients’ data could be obtained by eligible healthcare professionals. Briefly, each patient, either suspected of having a possible COVID-19 infection (e.g., with pyrexia, loss of taste, or flu-like symptoms, etc.), or a subject with a history of contact with a COVID-19-positive patient, could contact their family doctor and have a COVID-19 mRNA test ordered online through a state-run electronic referral system. The test could then be performed in all institutions who were registered to perform tests, and the result was then input into the central database. Subsequently, the test result, either positive or negative, was accessible by eligible healthcare professionals through the webpage listed above. In our case, all patients were recruited from a single GP practice (“Your Family Doctor”). The total observation period was extended for 12 months (till May 2021).

25OHD was measured by means of the Elecsys Vitamin D Total II assay, using a Cobas 801 analyzer with intra-assay variation of 1.1–3.1% and inter-assay variation of 2.2–4.3%.

25OHD sufficiency cut-offs were defined as 25OHD >20 ng/mL (50 nmol/L) [12] or as 25OHD > 30 ng/mL (75 nmol/L) [13,14]. The issue of whether a 20 ng/mL or 30 ng/mL cut-off should be applied for the Polish population was discussed in our previous study [15].

Statistical analysis: The MedCalc version 19.0.7 package was used for the statistical analysis. The D’Agostino–Pearson test was used to verify the normality of distribution. Data were compared using an independent samples *t*-test or Mann–Whitney test, respectively. The chi-square (χ^2^) test was also used. A *p* < 0.05 level was considered statistically significant.

This study was conducted according to the guidelines presented in the Declaration of Helsinki, and all procedures involving research study participants were approved by the Ethics Committee of the Polish Mother’s Memorial Hospital Research Institute, Lodz, Poland, decision nr 100/2019. Informed consent was obtained from all subjects involved in the study.

## 3. Results

The average 25OHD concentrations were 18.1 ± 7.37 ng/mL (median 17.0 ng/mL) and the average total calcium concentrations were 9.53 ± 0.41 mg/dL, and all were within the reference range for all subjects. The 25OHD concentrations were higher in spring in women (19.38 ± 7.60 ng/mL (median 19.0 ng/mL) versus 16.59 ± 6.82 ng/mL (median 15.1 ng/mL), *p* = 0.02)

The prevalence of 25OHD sufficiency, insufficiency or deficiency according to pre-defined cut-offs is presented in Table 1.

The analyses of cases of subsequent COVID-19 infections, stratified according to the initial 25OHD status, are presented in Table 2 and Table 3. All cases of COVID-19 infection in our study cohort were mild, mostly with moderate pyrexia and upper respiratory tract symptoms, and there were no deaths or hospital admissions. As all infected individuals were subjected to automatic state-imposed quarantine, it was not possible to assess the duration of infection. Patients with 25OHD concentrations below 12 ng/mL (30 nmol/L) in March and April 2020 were more likely to develop subsequent COVID 19 infection (11 out of 25 patients—44.0%, versus 20/109 patients—18.3%, *p* = 0.0063—Table 2). On the other hand, the difference was no longer significant for subjects with initial 25OHD concentrations below 20 ng/mL (50 nmol/L)—22 out of 84 (26.2%), versus 9 out of 50 (18.0%), *p* = 0.2787. This implies that the inclusion of patients with 25OHD insufficiency (12–20 ng/mL) according to the Institute of Medicine definition [12] rendered the difference to be no longer statistically significant.

## 4. Discussion

Our study demonstrates that only severe 25OHD deficiency at the beginning of COVID-19 infection was associated with an increased subsequent risk of developing an overt infection, although there were no cases of deaths or hospital admissions within our study group (i.e., healthy individuals aged 6–50). The study is valuable in the context that none of these individuals received any vitamin D supplements for the entire observation period, while the observation period encompassed the time before the start of the COVID-19 population vaccination program (health service workers in Poland had been vaccinated since January 2021, but the general population in this age group only since late spring 2021). Furthermore, the study included a COVID-19 antibody-naive population that is of particular importance, given that it will be no longer possible obtain such a study cohort, as by now, a great majority of the population has either been vaccinated or has suffered from COVID-19 infection.

Our results are consistent with the effects of the large prospective intervention study CORONAVIT (phase 3, open-label, randomized controlled trial) conducted in the UK, which showed that the supplementation of vitamin D in subjects with suboptimal vitamin D status (<30 ng/mL; *n* = 2674) increased 25OHD concentrations; however, this was not associated with protection against COVID-19 [16]. Our results are also in keeping with the results of the metanalysis by Wang et al. [17], where the authors, analyzing data from 16 studies (2756 patients), confirmed increased mortality, and an increased likelihood of hospital admissions and longer hospital stay, particularly in patients with more severe 25OHD deficiency (12 ng/mL cut-off was applied in seven studies, 20 ng/mL cut-off in eight studies, 25 ng/mL cut-off in one study, while a 30 ng/mL cut-off [13,14] was never applied). It should be noted, however, that several studies, e.g., [18,19,20], analyzed 25OHD in either hospitalized or symptomatic patients with COVID-19 infection, where these concentrations were indeed lower than in the general population or in age-matched controls. Furthermore, the potential benefits of vitamin D treatment in hospitalized patients (e.g., defined as a reduction in the need for Intensive Care Unit admission) [21] were not applicable to our cohort, as none of our patients required hospital admission. In such settings, caution must be applied to interpretation of the above data, since it is well recognized that 25OHD is an acute phase reactant, as there is a marked decrease in 25OHD as well as in vitamin D binding protein concentrations during an acute illness or any major stress (e.g., an elective surgery). Furthermore, lower 25OHD concentrations may persist for up to three months [22]. For instance, standard total hip replacement, even without any concomitant infection, is associated with around a 21–34% decrease in 25OHD levels [23]. Hence, some authors, e.g., Oscanoa et al. [24], warn about a possibility of “reverse causality” or ”backward causation”, which might have caused a significant bias in several studies on vitamin D and COVID-19 severity. In other words, “reverse causality” applies to a situation where an outcome seemingly determines the cause, thus leading to an erroneous conclusion where in fact an opposite relationship might exist. In this case, increased mortality/morbidity in COVID-19 patients might have been attributed to lower 25OHD concentrations, while a more severe course of COVID-19 infection (due to as-yet unidentified factors) might have resulted in lower 25OHD levels, as the consequence of a decline in 25OHD typical for any acute illness.

In our study, however, 25OHD concentrations were measured in healthy individuals without symptoms or signs of COVID-19 infections, where only a few cases of COVID-19 were noted in Poland in early spring 2020 (indeed, the first case of COVID-19 infection in Poland was announced on 4 March 2020). Hence, we can conclude that our study is free of potential “reverse causality” bias. We demonstrated that only subjects with severe 25OHD deficiency (<12 ng–30 nmol/L) were at an increased risk of subsequent COVID-19 infection, while it was no longer significant for concentrations above 20 ng/mL (50 nmol/L), i.e., a concentration considered sufficient by the Institute of Medicine [12]. In our previous study [15] we conclusively demonstrated that the majority of healthy individuals aged 6–50, in latitudes similar to Poland (49° to 54° N), obtain concentrations of 25OHD > 20 ng/mL in autumn without any supplementation, particularly if they have at least a two-week holiday period in summer. This observation clearly does not preclude the need for vitamin D supplementation in a selected group of subjects, e.g., in elderly obese individuals [25], or as an adjunct treatment in weight-loss programs [26]. Our cohort was, however, too small to assess any relationship between the risk of COVID-19 infection and 25OHD status stratified according to BMI quartiles. Interestingly, in the recent VITAL study [9], the authors questioned the value of vitamin D supplements in healthy subjects above 50. This was then summarized in the *New England Journal of Medicine* editorial [27], where the authors state that *“the lack of an effect for preventing numerous conditions suggests that providers should stop screening for 25-hydroxyvitamin D levels or recommending vitamin D supplements, and people should stop taking vitamin D supplements to prevent major diseases or extend life.”*

The limitations of our study include a relatively small sample size and recruitment from a single general practice, although this ensured completeness of the data and limited the potential drop-out rate, as all patients were managed by a single physician (MB-L). The one-year observation period was also a limitation, but this was related to the introduction of the universal COVID-19 vaccination program in Poland, which altered the relationship between vitamin D sta8tus and the potential risk of COVID-19 infection. Although our study was not designed to address the issue of vitamin supplementation, in view of our previous findings [15], we can suggest that among healthy individuals age 6–50 there is no need to take any 25OHD supplements for COVID-19 prevention if their 25OHD concentrations are above 20 ng/mL, while such levels can be successfully obtained through lifestyle measures.

## Figures and Tables

**Table 1:** Distribution of the study cohort (*n* = 134) with regard to vitamin D (25OHD) status, according to definition 1 [12]: 25OHD sufficiency > 20 ng/mL, insufficiency 12–20 ng/mL, deficiency < 12 ng/mL; or definition 2 [13,14]: 25OHD sufficiency > 30 ng/mL, insufficiency 20–30 ng/mL, deficiency < 20 ng/mL.

25OHD	Definition 1 [12]			Definition 2 [13,14]		
	Sufficiency > 20 ng/mL	Insufficiency 12.0–20 ng/mL	Deficiency < 12.0 ng/mL	Sufficiency >30 ng/mL	Insufficiency 20–30 ng/mL	Deficiency < 20 ng/mL
*n* = 134	50 (37.3%)	59 (44.0%)	25 (18.6%)	7 (5.2%)	43 (32.1%)	84 (62.7%)

**Table 2:** Distribution analysis of subsequent cases of COVID-19 infections in healthy subjects during 12 months observation period, stratified according to vitamin D (25OHD) concentrations above or below 12 ng/mL (30 nmol/L) in spring 2020. The difference is statistically significant, *p* = 0.0063, χ^2^ test.

COVID-19 Infection	25OHD < 12 ng/mL		
	NO	YES	
NO	89 (for > 12 ng/mL)	14 (for < 12 ng/mL)	103 (76.9%)
YES	20 (for > 12 ng/mL)	11 (for < 12 ng/mL)	31 (23.1%)
Total	109	25	134 (100%)

**Table 3:** Distribution analysis of subsequent cases of COVID-19 infections during 12 months observation period in healthy subjects, stratified according to vitamin D (25OHD) concentrations above or below 20 ng/mL (50 nmol/L) in spring 2020. The difference was not statistically significant, *p* = 0.2787, χ^2^ test.

COVID-19 Infection	25OHD < 20 ng/mL		
	NO	YES	
NO	41 (for > 20 ng/mL)	62 (for < 20 ng/mL)	103 (76.9%)
YES	9 (for > 20 ng/mL)	22 (for < 20 ng/mL)	31 (23.1%)
Total	50	84	134 (100%)

## Data Availability

The data presented in this study are available on request from the corresponding author.
